# The essential role of intestinal microbiota in cytomegalovirus reactivation

**DOI:** 10.1128/spectrum.02341-23

**Published:** 2023-09-27

**Authors:** Chikara Kohda, Satoshi Ino, Hiroki Ishikawa, Yoshihiro Kuno, Ryuichi Nagashima, Masayuki Iyoda

**Affiliations:** 1 Department of Microbiology and Immunology, Showa University School of Medicine, Tokyo, Japan; 2 Department of Medicine, Division of Nephrology, Showa University School of Medicine, Tokyo, Japan; University of Wisconsin-Madison, Madison, Wisconsin, USA

**Keywords:** murine cytomegalovirus, reactivation, latent infection, gut microbiota

## Abstract

Human cytomegalovirus (HCMV) is a member of Herpesviridae. It has been reported that HCMV is reactivated in the breast milk of HCMV-seropositive lactating women. As we have reported various aspects of the roles of indigenous microbiota, its role in the murine CMV (MCMV) reactivation was examined in this study. MCMV was latently infected in the salivary gland, mammary tissues, and colon in the pregnant mice. When the salivary gland, mammary tissues, and colon were removed 5 days after delivery, MCMV reactivation of latent infection in each organ was confirmed by the detection of MCMV IE1 mRNA using reverse transcription-quantitative PCR. MCMV reactivation was observed in 100% of the mice during pregnancy. Next, for the elimination of intestinal microbiota, the pregnant mice were treated with low-dose or high-dose non-absorbable antibiotics. Although the numbers of aerobe/anaerobe in cecal content in low-dose antibiotic-treated mice were comparable to those in untreated controls, high-dose antibiotic treatment decreased the number of aerobe/anaerobe microbes from ca.9.0 Log10 to ca.3.0 Log10 (cfu/g). However, it could not be confirmed in 16S rRNA analysis that specific bacterial phylum or genus was eliminated by this high-dose treatment. Interestingly, MCMV reactivation was also observed in 100% of low-dose antibiotic-treated mice, whereas, in high-dose antibiotic-treated mice, MCMV reactivation was not observed in the salivary gland or colon. MCMV IE1 mRNA was detected only in 33% of the mammary tissues of those high-dose-treated mice. These results suggest that the indigenous microbiota played a crucial role in the reactivation of latent infection.

**IMPORTANCE:**

Human cytomegalovirus (HCMV) infection via breast milk is a serious problem for very preterm infants such as developing a sepsis-like syndrome, cholestasis, or bronchopulmonary dysplasia, among others. It has been reported that HCMV is reactivated in the breast milk of HCMV-seropositive lactating women. In this study, the roles of indigenous microbiota in the murine CMV (MCMV) reactivation were examined using a mouse model. In MCMV latently infected mice, MCMV reactivation was observed in 100% of the mice during pregnancy. For the elimination of intestinal microbiota, MCMV-latent mice were treated with non-absorbable antibiotics. After delivery, MCMV reactivation was not observed in antibiotic-treated mice. This result suggested that the indigenous microbiota played a crucial role in the reactivation of latent infection.

## INTRODUCTION

Human cytomegalovirus (HCMV) is a double-stranded DNA virus of the family Herpesviridae. Humans are the only natural hosts of HCMV, and infections are common; the seroprevalence ranges from 45% to 100% (1). HCMV is more common in Asia, South America, and Africa than in Europe or the United States (1). Primary infection with HCMV, which usually occurs in childhood, is asymptomatic in a healthy person but can be life-threatening for the immune compromised, such as patients infected with human immunodeficiency virus (HIV) and organ transplant recipients. HCMV is also the leading cause of congenital viral infection. Congenital HCMV infection is usually associated with the occurrence of primary maternal infection during pregnancy or reactivation or re-infection with a different HCMV strain (2). The risk of congenital HCMV transmission is highest in a pregnant woman with no immunity who acquires primary HCMV infection during pregnancy (3, 4). In recent years, it was demonstrated that postnatal HCMV infection of very low birthweight (VLBW) infants through raw breast milk was increased (5 – 7). HCMV is reactivated in breast milk ranging from 42% to 97% of HCMV-seropositive lactating women. The reactivation of murine CMV (MCMV) is also observed in the pregnancy of the mouse (8). MCMV shares several hallmarks with HCMV, making it a useful model for examining viral infection within its natural host.

HCMV reactivation *in vivo* is well known, but the specific stimuli that lead to productive infection *in vivo* are still unclear. HCMV latency-associated transcripts have been also unclear (9 – 11). HCMV resides latently in monocytes, fibroblasts, myeloid cells, and endothelial cells (11, 12). HCMV can infect the entire gastrointestinal tract, and the colon is the most frequent site in immune-competent patients (13). Indeed, in ulcerative colitis patients, mucosal inflammation is often exacerbated by steroids. In these patients, steroids may induce HCMV colitis, resulting in worsening the symptoms.

In humans, the enormous kinds and amounts of microorganisms that inhabit mammalian body surfaces have a highly co-evolved relationship with the immune system. For example, there are reportedly approximately 1,000 kinds of enterobacteria, 100 trillion bacterial cells with approximately 0.5 million genes, and the mammalian immune system plays a fundamental role in maintaining homeostasis with resident enteromicrobiota. At the same time, indigenous enterobacteria profoundly shape host immunity. In previous work, Tanaka et al. demonstrated that the indigenous microbiota played a crucial role in the expansion and maintenance of MCMV-specific CD8^+^ memory T cells (14). Recently, it was reported that microbiota deviations were associated with an enhanced risk of atopic and diarrheal diseases (15, 16) and obesity (17 – 19). One mechanism here could lie in the ability of specific microbes to induce excessive energy harvest and storage (18). The importance of this process culminates in pregnancy. It previously investigated the role of gut microbiota on blood pressure during early pregnancy (20) and showed that systolic blood pressure was inversely correlated with the abundances of the butyrate-producing bacterium Odoribacter and the Clostridiaceae family (20).

To date, there are limited studies on the effects of gut microbiota composition on the reactivation of HCMV. The present study aims to clarify whether enterobacteria participate in CMV reactivation. Therefore, we examined the reactivation of latent MCMV infection in the pregnant mice of the salivary gland, mammary tissues, and colon by antibiotics treatment using reverse transcription-quantitative PCR (RT-qPCR).

## RESULTS

### The reactivation of MCMV occurs in pregnancy

**Fig 1:**
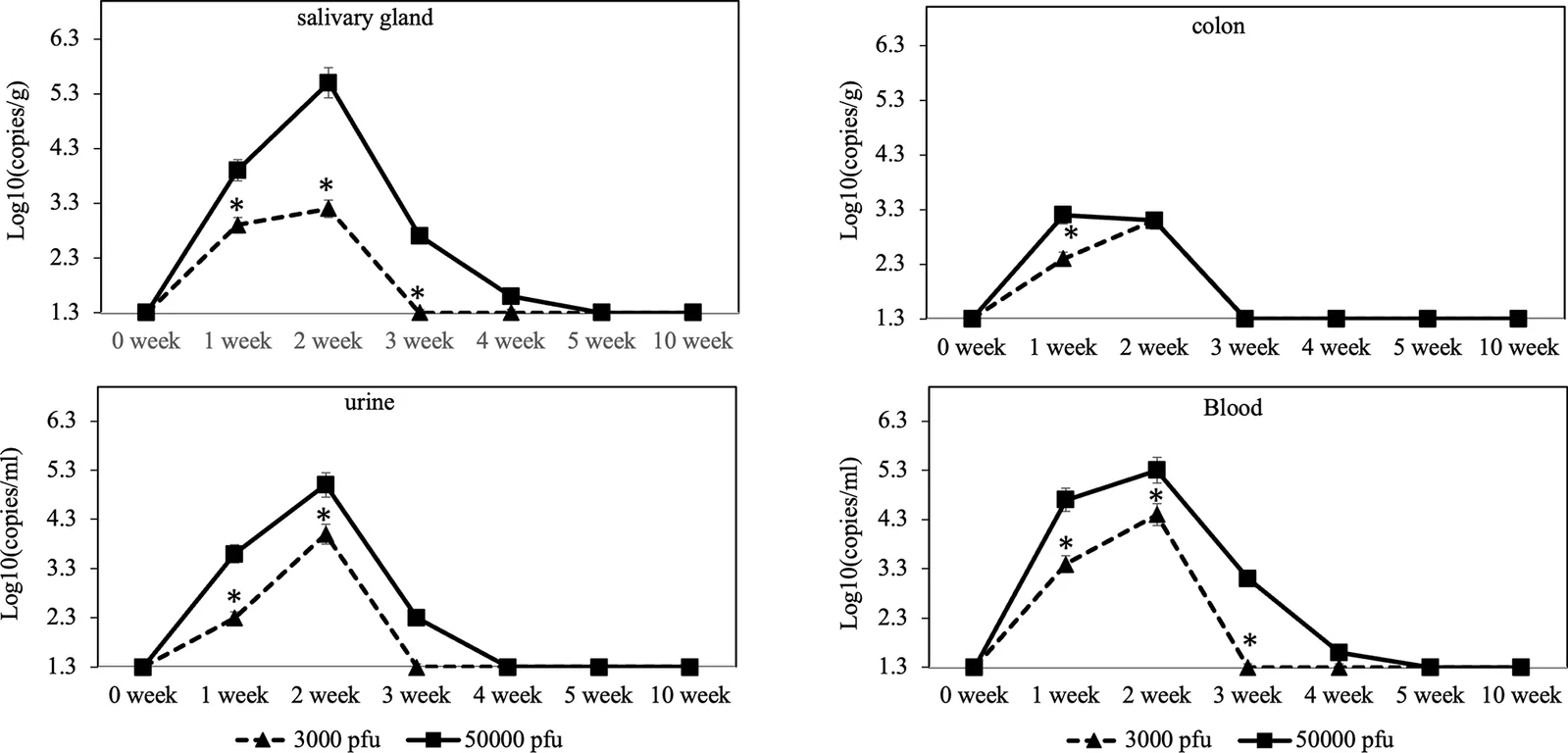
MCMV infected various tissues in Balb/c mice. Balb/c mice were administered 3,000 and 50,000 PFU of MCMV via i.p. injection. IE1 mRNA copy numbers were assessed in salivary gland, colon, urine, and blood by RT-qPCR. Five mice were analyzed at each time point. An asterisk (*) shows a significant difference compared to high-dose MCMV-infected mice (*P* < 0.05).

**Fig 2:**
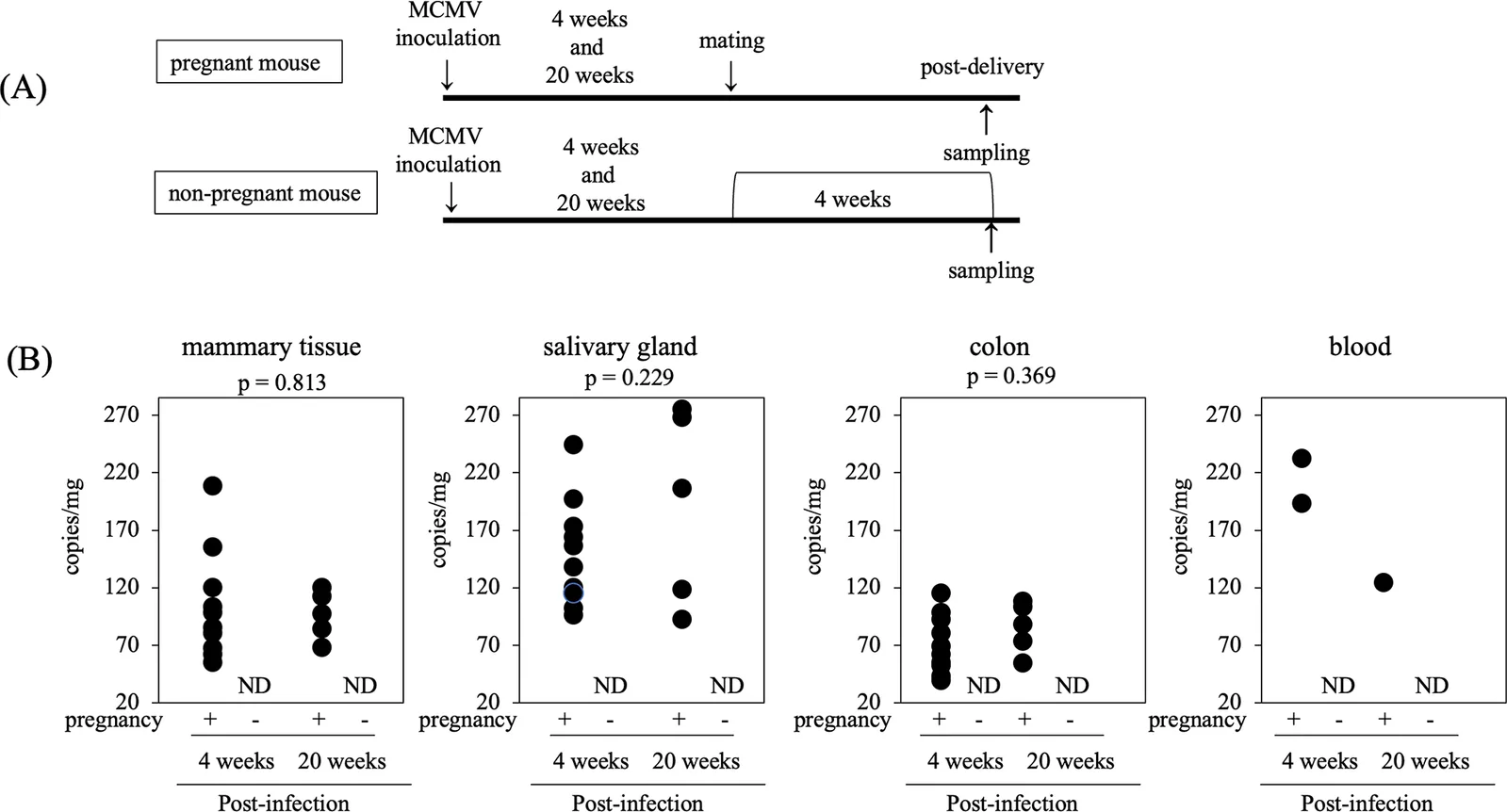
Reactivation of MCMV in the pregnancy. IE1 mRNA copy numbers in the salivary gland, mammary tissues, colon, and blood obtained from mothers. The organs were removed after the fifth delivery, and then the RNA was extracted. RT-qPCR was done by hydrolysis probe method. (**A**) The experimental design in MCMV reactivation study. (**B**) Mice were pregnant at 4 weeks and 20 weeks after MCMV inoculation, and expression of IE1 mRNA was compared in each organ.

**Fig 3:**
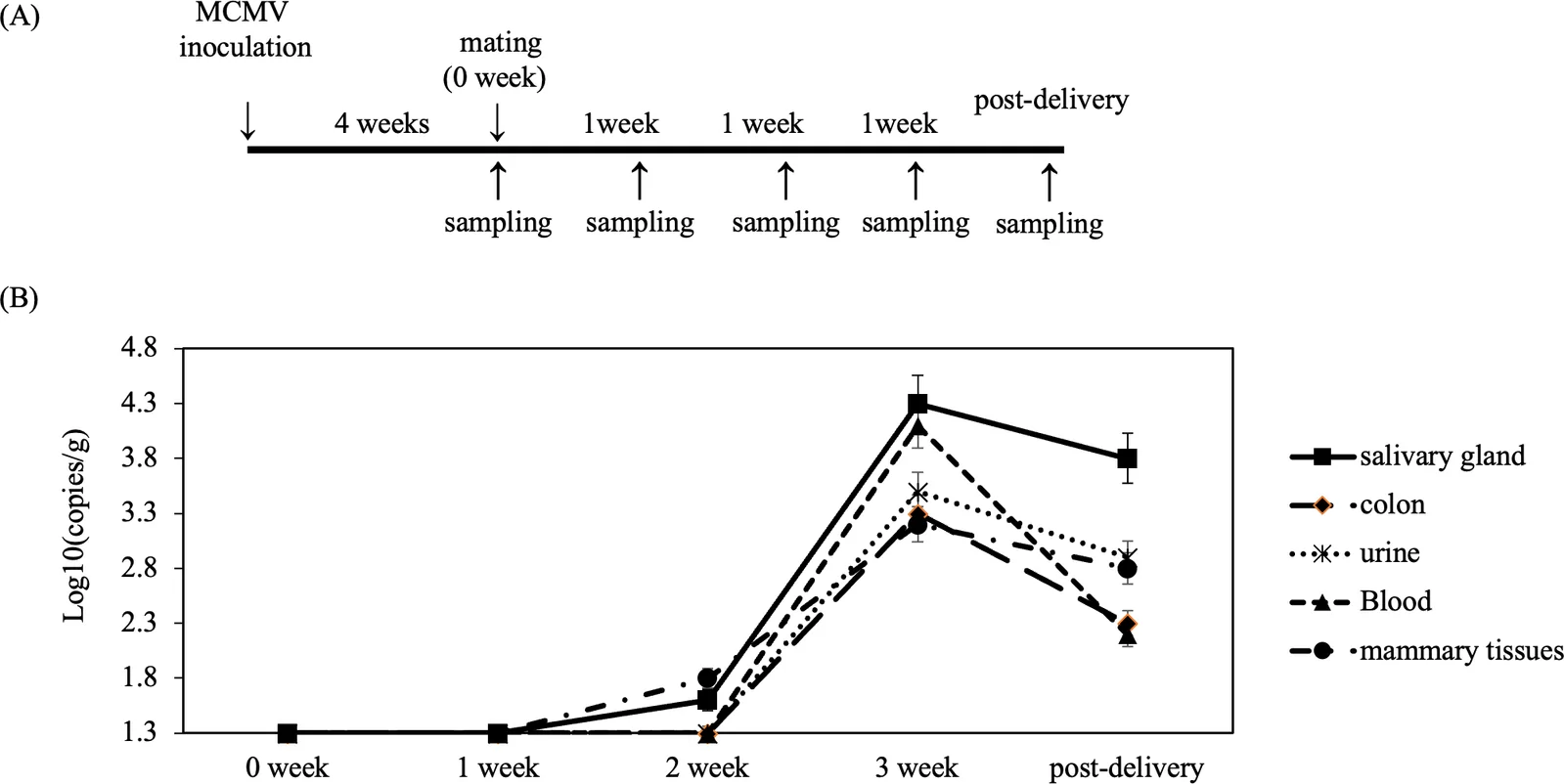
The time point of MCMV reactivation during pregnancy. (**A**) Experimental design to assess the time point of MCMV reactivation. (**B**) MCMV IE1 mRNA copy numbers were measured at 0, 1, 2, and 3 weeks of gestation and after delivery.

At first, we investigated the timing of the transition to MCMV latent infection. Female Balb/c mice were infected with 3,000 and 50,000 PFU of MCMV strain Smith by intraperitoneal (i.p.) injection and were observed kinetics of MCMV infection in various organs. Following i.p. infection of BALB/c mice with MCMV at a low viral dose of 3,000 PFU, the virus titers peaked by week 2 and became undetectable by week 3 (Fig. 1). The detection limit was Log10 (cfu/g) and Log10 (cfu/mL) = 1.3. On the other hand, with the mouse which inoculated 50,000 PFU, MCMV was detected in the blood and salivary gland of 20% (1/5) of mice after the virus inoculation for 4 weeks (Fig. 1). Because a virus was not detected 3 weeks after the MCMV inoculation, it was suggested that MCMV transitioned from acute to latent infection 3 weeks later by a low-dose infection. Next, MCMV IE-1 mRNA was detected from each organ on the fifth day after birth. At 4 weeks post-inoculation, there might be low, undetectable levels of viral replication, so comparisons were made with MCMV-latent mice at 20 weeks post-inoculation (Fig. 2A). MCMV IE-1 mRNA was detected in the salivary gland, mammary tissues, colon, and blood from pregnant mice but was not detected in non-pregnant mice (Fig. 2B). This result indicated that MCMV reactivation and replication could occur during pregnancy or shortly after delivery. As for the MCMV titer detected in each organ, a significant difference was not recognized between the mouse group with 4 weeks and 20 weeks post-inoculation (Fig. 2). From this result, it was decided that the mouse with MCMV infection waited at least 4 weeks to establish viral latency. Then, we decided on the time point of MCMV reactivation during pregnancy. As shown in Fig. 3, MCMV IE-1 mRNA was detected in the salivary gland and mammary tissues from mice of the pregnancy second week, and was detected in the salivary gland, mammary tissues, colon, and blood from all mice of the pregnancy third week. In this experiment, MCMV IE-1 mRNA was detected in 20% (1/5) of mice in the second week of pregnancy. These results revealed that reactivation of MCMV occurred from the second to the third week of pregnancy. In addition, the possibility that the virus spread hematogenously was thought about because MCMV was detected in the pregnancy’s third week by the blood of all mice.

### Transmission of infectious virus from latent mothers to breastfed neonates

**Fig 4:**
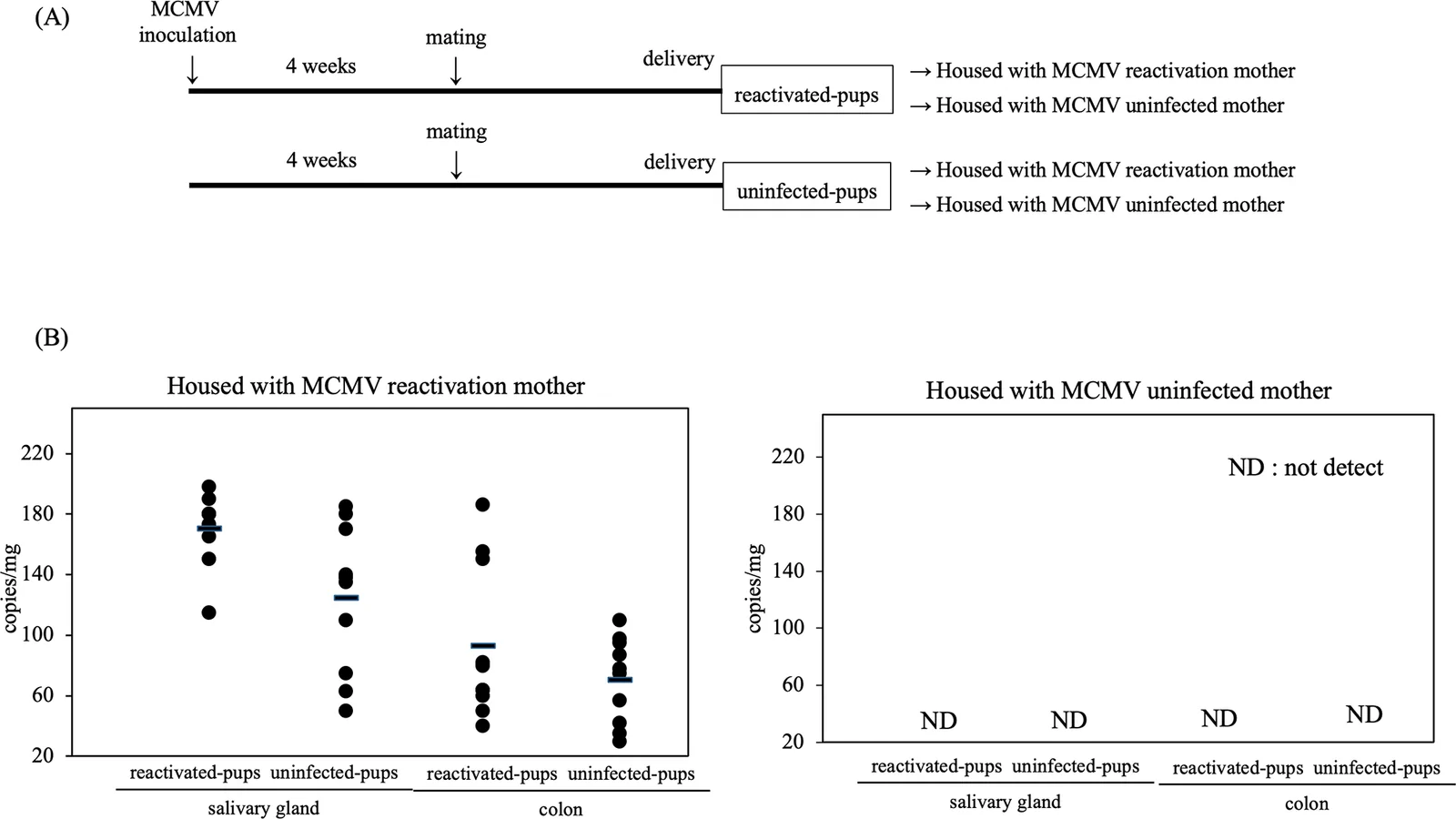
Infectivity of reactivated CMV. (**A**) Experimental design for CMV infectivity assessment. (**B**) Reactivated MCMV was transmitted to offspring. IE1 mRNA copy numbers in salivary gland, and colon obtained from reactivated and uninfected pups. The organs were removed after 14 days of breeding, and then the RNA was extracted. RT-qPCR was done by hydrolysis probe method.

Next, we confirmed the infectiousness of reactivation MCMV. The pups from the MCMV-reactivation mother were termed “reactivated pups,” whereas the pups from the MCMV-uninfected mother were termed “uninfected pups.” Reactivated pups and uninfected pups were housed with MCMV reactivation and uninfected mother for 14 days. As shown in Fig. 4, MCMV IE-1 mRNA was detected in the salivary gland and colon from all reactivated pups (*n* = 10) and uninfected pups (*n* = 10) housed with MCMV-reactivation mother but not in both pups housed with MCMV-uninfected mother. MCMV infection of pups housed with MCMV-reactivated mothers can be thought of as vertical transmission from MCMV-reactivated mothers and horizontal transmission among pups. Combined with the fact that MCMV IE1 mRNA was not detected in pups housed with MCMV-uninfected mothers, it was at least confirmed that vertical transmission from MCMV-reactivated mother to pup occurred. Expression of MCMV IE-1 mRNA does not necessarily indicate the production of an infectious virus, as abortive MCMV infection may exhibit a similar outcome. So, we utilized a median tissue culture infectious dose (TCID50) assay. Four MCMV-reactivation mothers nursed or fostered a total of 10 reactivated pups and raised 10 uninfected pups for 14 days. Organ homogenates were prepared from the salivary gland and colon of breastfed neonates and used to inoculate monolayers of 3T3 cells, which were observed daily for 3 weeks for the presence of cytopathic effect (CPE). As summarized in Table 1, infectious virus was detected in at least one organ, including the salivary gland (10 of 10 reactivated pups) and colon (10 of 10 reactivated pups), in 10 of 10 breastfed neonates and detected in the salivary gland (9 of 10 uninfected pups) and colon (9 of 10 uninfected pups). Importantly, all MCMV-reactivation mothers could transmit infectious viruses to their breastfed reactivated pups and uninfected pups.

**TABLE 1:** Detection of infectious MCMV in organ homogenates

The value of TCID50	No. of reactivated pups with positive MCMV vs no. of reactivated pups examined		No. of uninfected pups with positive MCMV vs no. of uninfected pups examined	
	Salivary gland	Colon	Salivary gland	Colon
0	0/10	0/10	1/10	1/10
10	9/10	10/10	9/10	9/10
100	1/10	0/10	0/10	0/10

### Change in the internal environment by the antibiotic treatment

**Fig 5:**
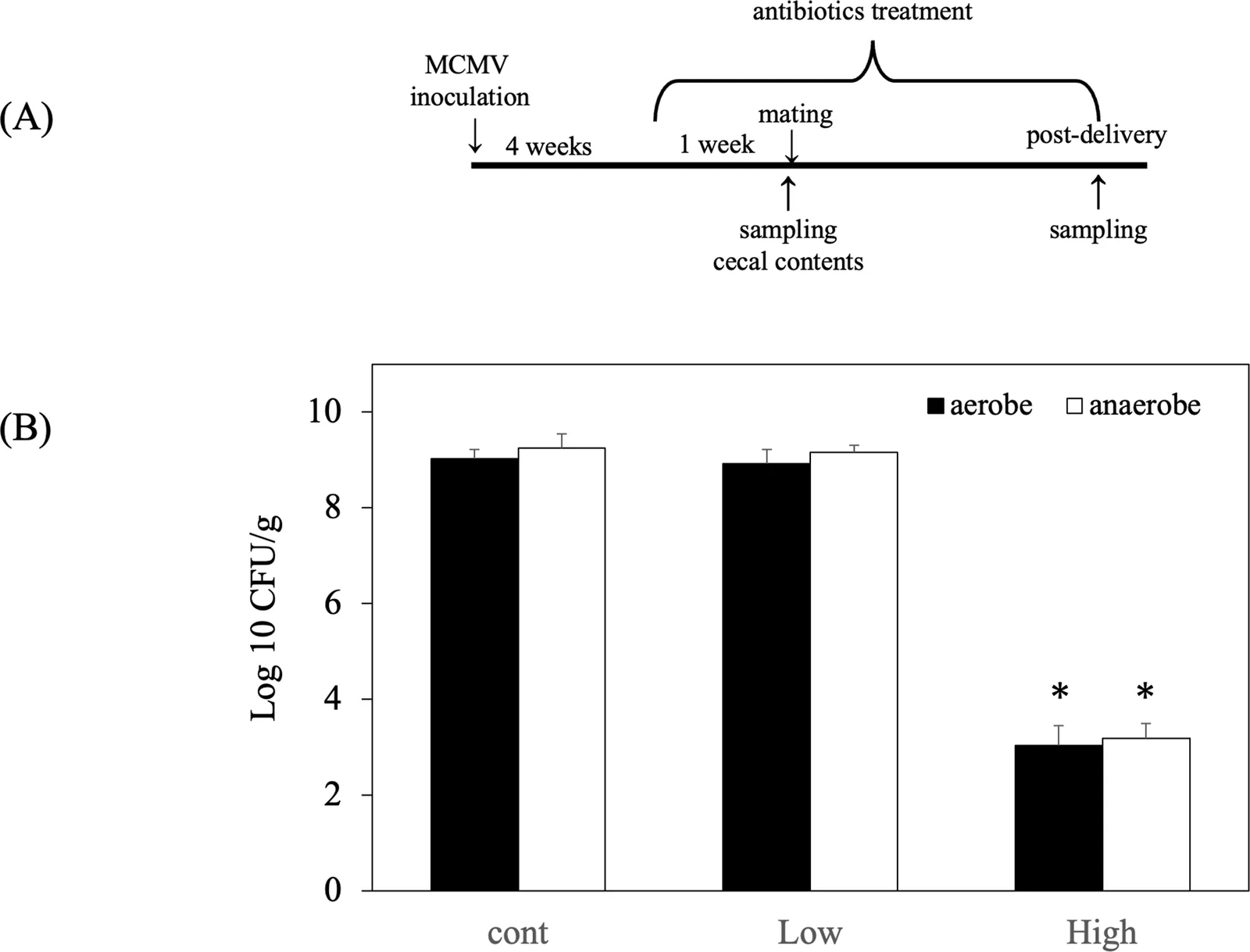
Effect of antibiotic treatment on viable counts of aerobic and anaerobic microbes in the cecum. (**A**) Experimental design of antibiotic treatment study. (**B**) After 5 days of antibiotic administration, the viable aerobes (■) and anaerobes (□︎︎︎) in cecal contents were determined. An asterisk (*) shows a significant difference compared to untreated mice (*P* < 0.05).

The change in the internal environment was evaluated by measuring the number of living microbes and analysis of the microbial population. In untreated control groups, approximately 9.22 log10 cfu/g aerobe and 9.28 log10 cfu/g anaerobe were cultured from the cecal content samples. To eliminate intestinal microbiota, we used four non-absorbable antibiotics that reach the large intestine without being absorbed in the small intestine. In comparison with a control group, the viable counts of microbes were maintained at the same level after low-dose antibiotic treatment. However, the levels of aerobically and anaerobically cultured microbes in high-dose administration groups were significantly decreased by the antibiotics treatment (Fig. 5).

**Fig 6:**
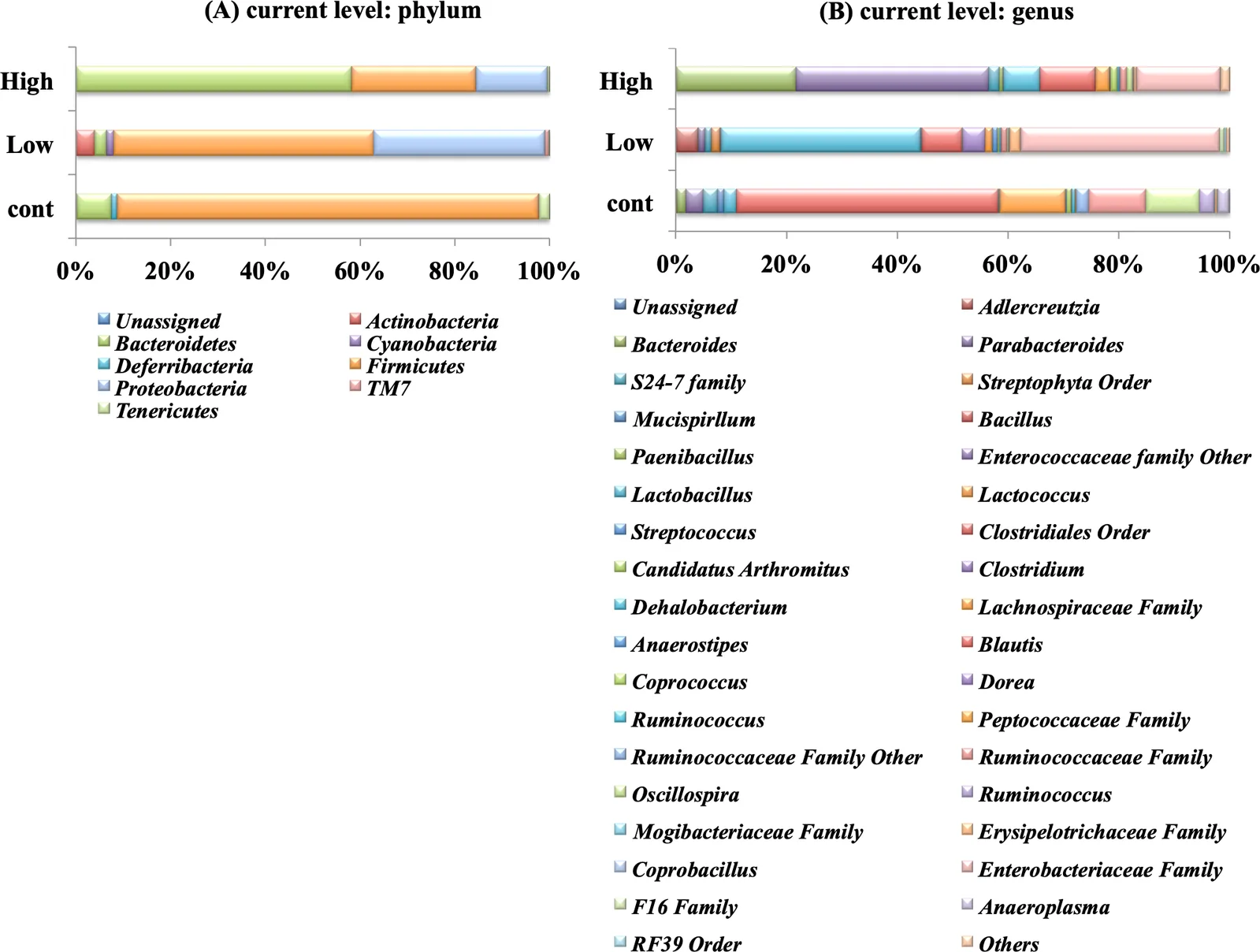
Microbial population in the cecum after 5 days of antibiotic treatment. Microbial population was different between the antibiotic-treated groups and control groups (*n* = 5 mice per group) evaluated by qPCR with universal primers for 16S rRNA. A stacked bar graph of specimens showed a relative abundance of bacteria at ≥0.1% at the phylum (**A**) and genus (**B**) taxonomic levels.

The analysis of 16S rRNA sequences was used to characterize microbial communities. 16S rRNA sequences, the small unit of ribosomal RNA in prokaryotes, are the most widely used sequences for inferring the phylogenetic relationship among microbial species. Operational taxonomic units (OTUs), usually defined as clusters of similar 16S rRNA sequences, are the most widely used basic diversity units in large-scale characterizations of microbial communities. After antibiotics treatment, the cecal microbiota changed in both groups (Fig. 6). In the untreated control groups, the two most common OTUs detected were in the phyla Bacteroidetes and Firmicutes. In the genus- or family-level community of bacteria, the three most common OTUs detected were in Clostridiales order (47.2%), Lachnospiraceae family (11.9%), and Ruminococcaceae family (10.4%).

In low-dose administration groups, the cecal microbiota was dominated by phylum Proteobacteria accounting for 36.1% of all microbes present, compared to 0.0% in the untreated controls. The phylum Firmicutes levels were lower in the antibiotic-treated mice than in the untreated controls (54.6% vs 88.9%). In the genus-level community of bacteria, the two most common OTUs detected were in the genus Lactobacillus (36.0%) and the family Enterobacteriaceae (35.9%).

In high-dose administration groups, the cecal microbiota was dominated by phylum Bacteroidetes accounting for 58.3% of all microbes present, compared to 7.3% in the untreated controls. The phylum Firmicutes levels were lower in the antibiotic-treated mice than in the untreated controls (26.3% vs 88.9%). The phylum Proteobacteria levels were higher in the antibiotic-treated mice than in the untreated controls (15.2% vs 0.0%). In the genus-level community of bacteria, the three most common OTUs detected were in the genera Bacteroidetes (21.6%) and Parabacteroides (34.8%), and the family Enterobacteriaceae (15.1%).

**Fig 7:**
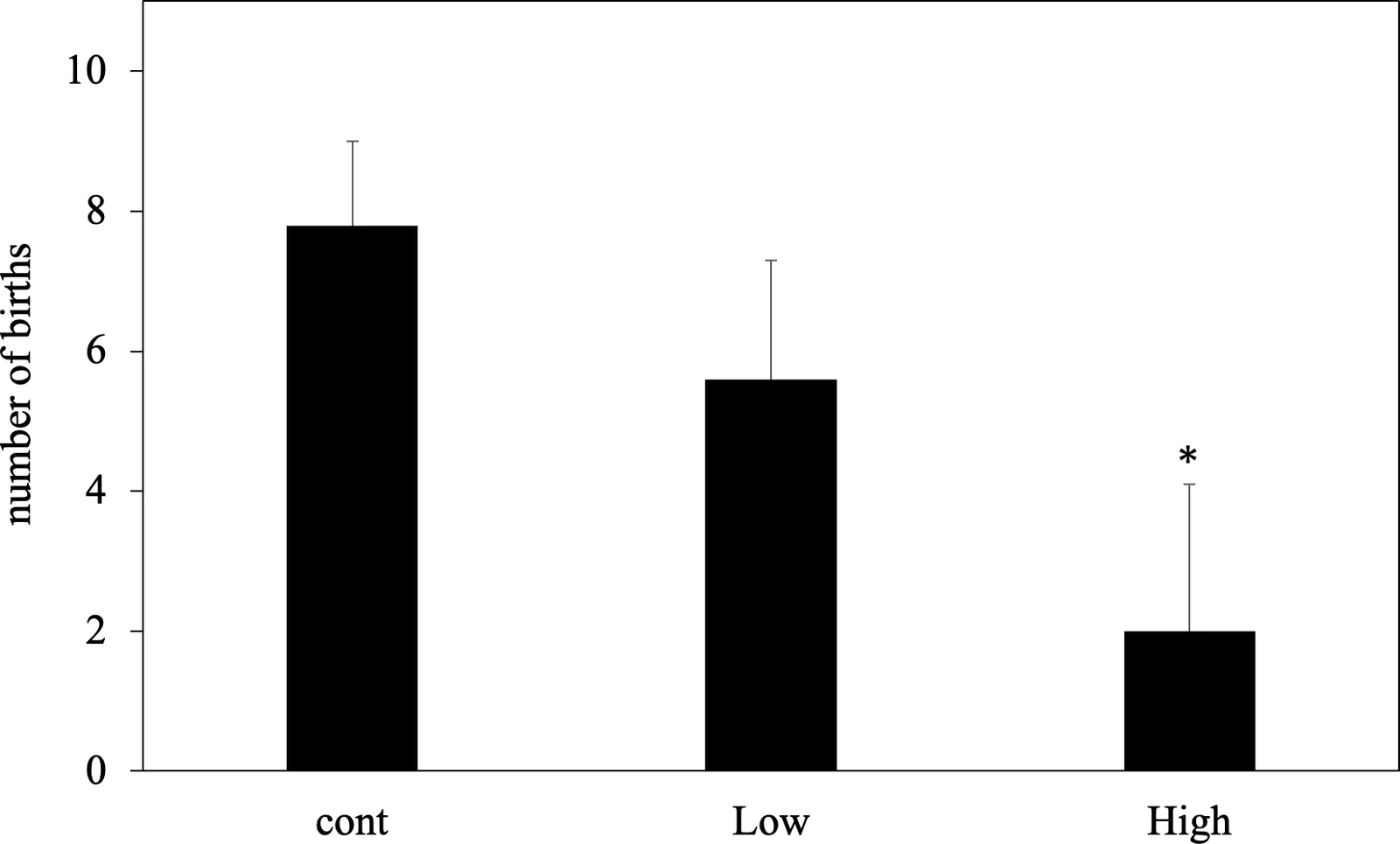
The number of offspring a mouse produced at first birth. An asterisk (*) shows a significant difference compared to untreated mice (*P* < 0.05).

With antibiotic-treated mothers, there was a small number of pups. Particularly, the number of pups was significantly decreased in high-dose administration groups (Fig. 7). This result might also be due to changes in the intestinal environment.

### MCMV reactivation was inhibited by a reduced abundance of intestinal bacteria

**Fig 8:**
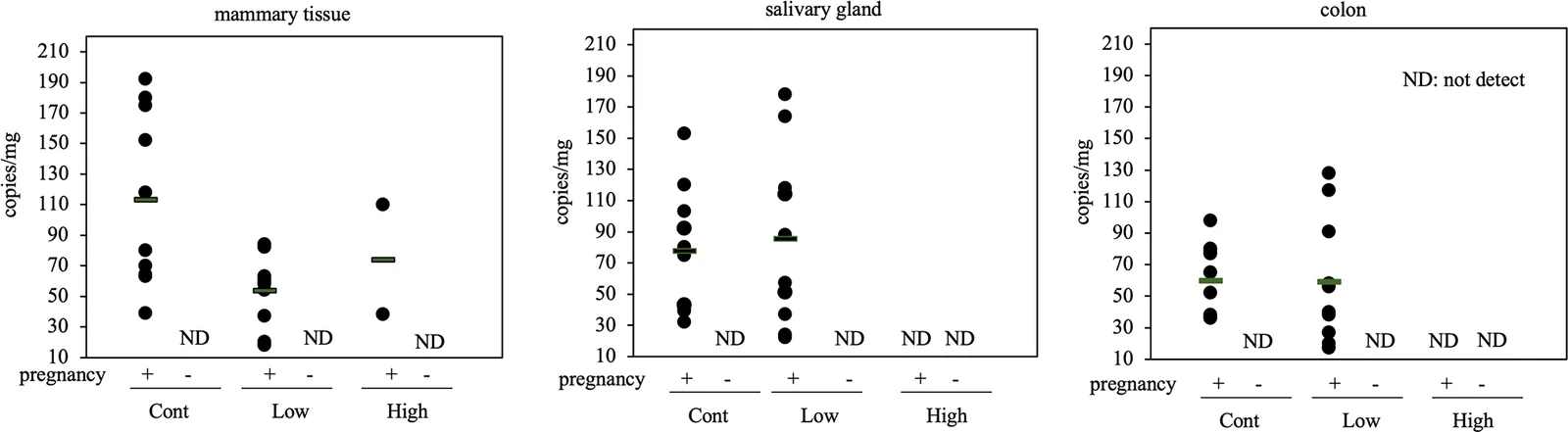
MCMV IE1 mRNA copy numbers in the salivary gland, mammary tissues, and colon were obtained from mothers and non-pregnant MCMV-latently infected mice. The organs were removed from mothers after fifth delivery and were removed from non-pregnant MCMV latently infected mice after 1 month of antibiotic treatment. RNA was extracted, and RT-qPCR was done by hydrolysis probe method.

**Fig 9:**
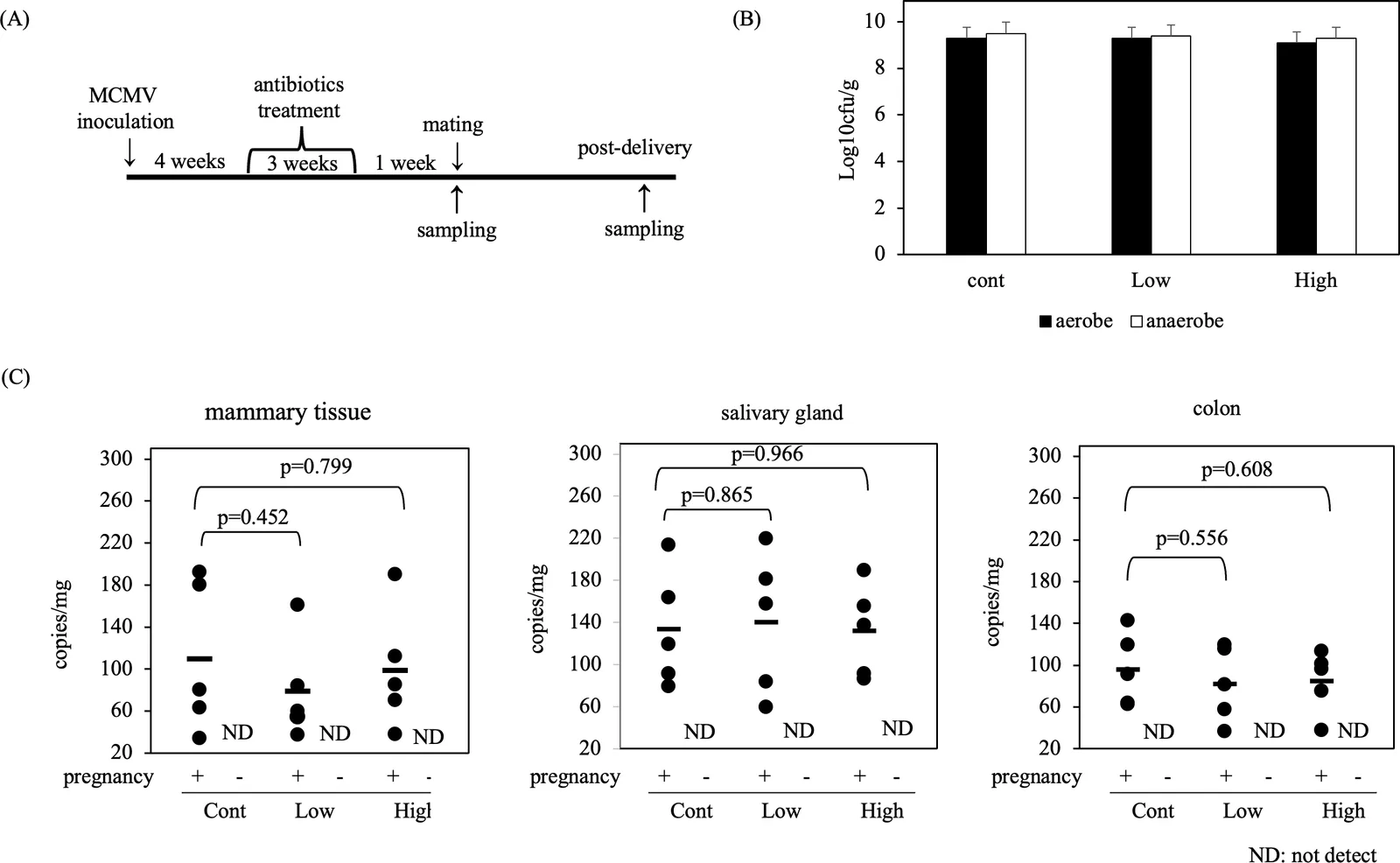
Recovery of MCMV reactivation with the restoration of viable counts of intestinal bacteria. (**A**) The experimental design in the recovery of MCMV reactivation study. (**B**) Effect of canceling the antibiotic treatment on viable counts of aerobic and anaerobic microbes in the cecum. After 1 week of canceling the antibiotic administration, the viable aerobes (■) and anaerobes (□︎) in cecal contents were determined. (**C**) Effect of canceling the antibiotic treatment on MCMV reactivation. MCMV IE1 mRNA copy numbers in the salivary gland, mammary tissues, and colon obtained from mothers. The organs were removed from mothers after fifth delivery.

The establishment of CMV reactivation was verified by the detection of MCMV IE1 mRNA in the salivary gland, mammary tissues, and colon during pregnancy or shortly after childbirth. After a delivery, MCMV IE1 mRNA was detected in all untreated mothers in the mammary tissues, salivary gland, and colon (Fig. 8). In low-dose administration groups, CMV IE1 mRNA was detected with all mice in the salivary gland and colon, but CMV IE1 mRNA detected in mammary tissues was 40% of mice (4/10). In high-dose administration groups, CMV IE1 mRNA was not detected in the salivary gland and colon, but CMV IE1 mRNA was detected in 33% (2/6) of mice in mammary tissues. After antibiotic treatment, MCMV IE1 mRNA was not detected in the mammary tissues, salivary gland, and colon from latently infected mice that were not pregnant (Fig. 8). It was shown that the use of the antibiotic caused a change in the gut microbiota, a more inappropriate or surplus drug dosage that the gut microbiota which changed could reduce the influence by canceling the use of the antibiotic (21). Therefore, we experimented on the MCMV reactivation using the mouse, and the viable counts of microbes were restored. As for the viable counts of microbes in the cecum, the levels of aerobically and anaerobically cultured microbes in high-dose administration groups were restored to the same level of untreated control groups after canceling the antibiotics treatment (Fig. 9B). Reactivation of the virus was observed when pregnant mice were withdrawn from the antibiotic treatment. No significant difference was observed in the MCMV mRNA expression level, which had recovered to the control level (Fig. 9C).

**Fig 10:**
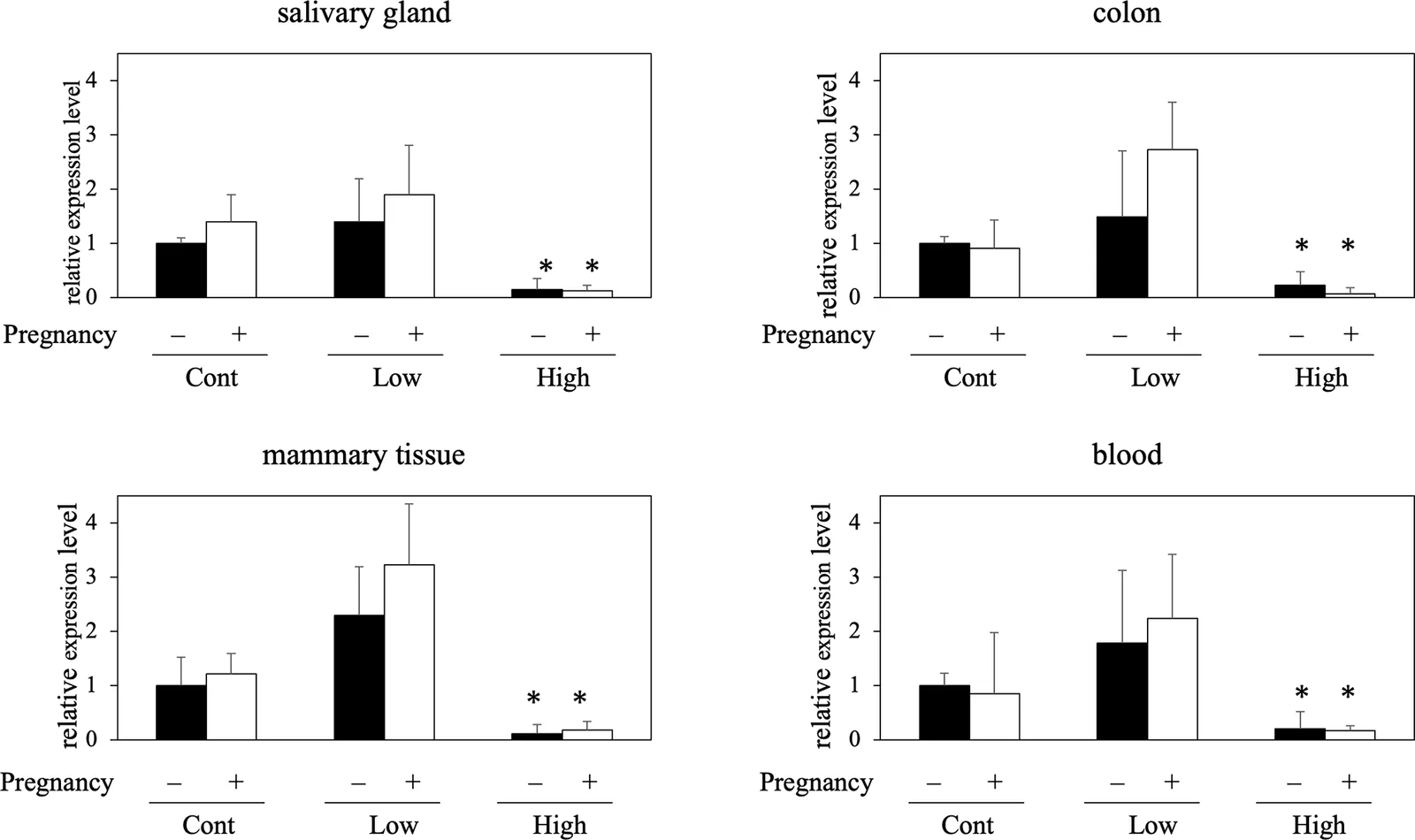
Expression levels of TNF-α mRNA in blood, salivary gland, mammary tissues, and colon at 5 days postpartum. The total RNA was extracted, and cDNA was synthesized. This material was a template in the RT-qPCR. TNF-α mRNA expression level of antibiotics-untreated non-pregnant mice was converted to 1. An asterisk (*) shows a significant difference compared to antibiotics-untreated non-pregnant mice (*P* < 0.05).

It was known that tumor necrosis factor alpha (TNF-α) triggers the reactivation of latent CMV (22, 23). Therefore, we measured TNF-α gene expression in each organ. The total cellular RNA was extracted, and TNF-α-specific mRNA expression was examined by RT-qPCR. In untreated control mice, the expression of TNF-α mRNA was shown to be constant after 5 days from parturition. In low-dose administration groups, the expression of TNF-α mRNA was increased in the mammary tissues, salivary gland, colon, and blood. In high-dose administration groups, the expression of TNF-α mRNA was significantly decreased in all samples (Fig. 10).

## DISCUSSION

In the present study, we showed that the MCMV reactivation was effectively inhibited by a reduced abundance of intestinal bacteria. It was clinically well known that HCMV reactivation was triggered through pathways activated by inflammation, infection, immunosuppression, injury, and pregnancy (24, 25). It would be of great interest to determine whether the finding that the gut microbiota was involved in CMV reactivation presented in this study was pregnancy specific or general. However, to date, this could not be confirmed because no mouse model reliably reactivates MCMV other than pregnancy.

In constructing a mouse model of MCMV reactivation, we determined the time for acute MCMV infection to transition to latent infection. Balb/c mice were infected with a high dose and a low dose of MCMV and observed the kinetics of the virus in the salivary gland, urine, blood, and colon. In the 3,000 PFU inoculation group, MCMV became less than a detection limit level from 3 weeks after MCMV inoculation. There were some reports of experiments with latent infection of MCMV (8, 26). Their reports showed that some waited >4 weeks to assess latent infection, but these were infected with high doses of 10^4^, 10^5^, or higher. Mice 4 weeks after CMV inoculation might have a low level of viral replication. However, in recent years, it has been reported that CMV had a subclinical infection that repeated latent infection and viral reactivation without clinical symptoms (27), so it was possible that mice 20 weeks after CMV inoculation might have a low level of viral replication. So, we compared the expression level of MCMV IE1 mRNA between the mouse group at 4 weeks and 20 weeks post-inoculation. As for the MCMV IE1 mRNA detected in each organ, a significant difference was not recognized between the mouse group at 4 weeks and 20 weeks post-inoculation. Intestinal bacteria would be described later, but it was observed that the older the mice treated with antibiotics, the less likely they were to become pregnant (data not shown). For these reasons, MCMV-infected mice housed for 4 weeks were considered to have latent MCMV infection in this study.

Next, we confirmed that MCMV reactivation occurred in pregnancy. To distinguish genomic DNA from active viral transcription, we detected MCMV IE-1 mRNA in the salivary gland, mammary tissues, and colon from mothers latently infected with MCMV. MCMV-IE1 mRNA was detected in the salivary gland, mammary tissues, and colon from MCMV-latent mothers. This observation suggested that MCMV reactivation occurred in pregnancy. Furthermore, it was clarified that this reactivation occurs from the second to the third week of pregnancy. As for HCMV, the virus latently infects mainly cells of the myeloid lineage. Latent viral genomes can be detected in peripheral monocytes (28) and traced back to their CD34+ progenitors in the bone marrow (29, 30). There is no evidence of virus carriage in peripheral blood B or T cells, whereas CD34+ bone marrow progenitor cells are a source of cells of the lymphoid lineage. Our results also indicated that CMV reactivation and replication could occur throughout the body, but we were unable to clarify where reactivation occurs and how it spreads throughout the body. By the same criterion, we observed transmission of MCMV from MCMV-reactivated mothers to nursing neonates or uninfected mice in the cage, with viral gene expression detected in the salivary gland and colon. Vertical transmission from MCMV-reactivated mother to offspring was confirmed by the presence of infectious MCMV in the salivary gland and colon of nursing pups. This result, together with the results of the TCID50 assay for MCMV in organs, indicated that reactivated MCMV was infectious and suggested that reactivation of latent MCMV was similar to human conditions of HCMV reactivation.

In this study, the mice treated with high-dose antibiotics had less offspring than controls or mice treated with low-dose antibiotics. This observation suggested that high-dose antibiotics may have non-specific impacts on the overall health of the mice which may negatively influence MCMV replication. However, it has been reported that germ-free mice have fewer offspring than conventional mice (31), and reproductive ability in germ-free mice remains inferior to their conventional counterparts even after improvement of feed and other such rearing conditions (32). Based on these findings, we considered that the absence of MCMV reactivation and the decrease in the number of births in the high-concentration administration group were due to the effects of intestinal bacteria, not the effects of antibiotics.

The gut microbiota comprises an enormous collection of microbes. In mice, it is dominated by two major bacterial phyla, Bacteroidetes and Firmicutes. It was shown that the microbial population was similar to our mouse model. In low-dose administration groups, although the microbial constitution was altered, the viable counts of microbes were maintained at the same level as those in an untreated group. After low-dose antibiotic treatment, the phylum Firmicutes levels were lower in the antibiotic-treated mice than in the untreated controls. This intestinal condition was thought to be “dysbiosis.” In this condition, CMV reactivation occurred during pregnancy with all mice, but viral reactivation in the mammary tissues was not recognized in some mice. On the other hand, the phylum Firmicutes level and the viable counts of microbes were significantly decreased by the high-dose antibiotic treatment. In this bacterial-reduced condition, CMV reactivation was inhibited in all mice. Furthermore, MCMV reactivation occurred by reconstitution of the gut microbiota after antibiotics cessation. There were several reports on the intestinal microbiota after antibiotics cessation, and it was known that microbial communities began to return to their initial state, but the return was often incomplete (32, 33). In this study, it was suggested that the gut microbiota had returned to the initial state before the administration of antibiotics. Although the crucial mechanism of indigenous microbiota remains unclear, these data suggest that the indigenous microbiota played a crucial role in the reactivation of latent infection. The identification of groups of microorganisms responsible for CMV reactivation is underway; furthermore, individual functional molecules or gut microbial metabolites may be identified. This is a future consideration.

The host immune responses control both pathogenic latent viruses and bacteria. However, it is unclear whether various immune mediators, such as certain cytokines and chemokines, exert primarily a protective or destructive role in viral reactivation. Also, some immune mechanisms that are active against viruses may diminish antibacterial immune responses and vice versa. It was reported that TNF-α induced CMV reactivation in a transfected human monocytic cell line. We thus measured the expression level of TNF-α mRNA in each organ. TNF-α was involved in viral reactivation because TNF-α mRNA expression was significantly lower in the high-dose antibiotics-treatment group, in which MCMV reactivation was not observed. However, the similarity of TNF-α mRNA levels in MCMV-reactivated and non-reactivated non-pregnant mice makes this unlikely to be a trigger. It was suggested that TNF-α may need to be maintained at a certain concentration or higher during MCMV reactivation.

It is well established that the intestinal microbiota influences host metabolism, nutrient absorption, and immune function and that disruption of this balanced community can have very serious health implications (34 – 36). Important mediators of these interactions can be microbial metabolites. These are small, diffusible factors capable of engaging host cells, which could facilitate their ability to modulate basic physiologic processes (37). Specific molecules influence important aspects of the development of immune cell subsets (38 – 40). It was demonstrated that microbiota-derived butyric acid could reactivate the latently infected HIV-1 and Epstein-Barr virus (41, 42). As we mentioned above, high levels of the phylum Firmicutes existed in antibiotic-untreated control mice. Clostridia included in the phylum Firmicutes was known to produce short-chain fatty acids (especially for butyrate). Butyrate is the primary energy source for colonocytes and functions as a histone deacetylase inhibitor (43). Because histone acetylation was known to control the expression of the CMV lytic gene (44), butyrate may participate in the reactivation of CMV. However, how specific microbiota-derived signals directly influence CMV reactivation remains still unknown. In addition, the viral reactivation in our mouse model depended on a pregnancy, complex mechanism. However, the results obtained in this study demonstrated that intestinal microbiota affected the MCMV latency. In addition, manipulation of the microbiota induced MCMV reactivation presumably through suppression of TNF-α production.

HCMV infection may be acquired perinatally or prenatally and is the most common congenital viral infection. In addition, it was demonstrated that postnatal HCMV infection of VLBW infants through raw breast milk was increased. Symptoms of this HCMV infection in VLBW infants include microcephaly, jaundice, petechiae, hepatosplenomegaly, pneumonitis, hepatitis, and sensorineural hearing loss. Given the significant impact of CMV infection on health care, there are still no effective vaccines and fundamental therapies. Although the relative contribution of virus reactivation or re-infection to congenital infections is not clear, prevention of infection in either scenario would be beneficial. The significance of our research is in understanding the mechanism for viral re-infection, which will allow the development of new medical technologies such as the prevention of CMV infection by controlling intestinal flora and treatment targeting CMV latently infected cells. This would be the first report to discuss the role of microbiota in CMV reactivation.

## MATERIALS AND METHODS

### Mice and infection

BALB/c female mice (Oriental Yeast Co., Ltd., Tokyo, Japan) were used for experiments. The protocol used in the present study was approved by the Institutional Animal Care and Use Committee of Showa University. Stocks of MCMV strain Smith (ATCC VR-1399) were used, and virus titers were determined by standard plaque assays. For MCMV infection, 5-week-old mice were infected with the MCMV Smith strain, via i.p. inoculation with 3,000 PFU. Infected mice housed for 4 weeks were considered to have latent MCMV infection. The establishment of latency was verified by the absence of detectable infectious viruses in the urine.

### Antibiotics treatment

Antibiotic treatment was carried out as described previously with minor modifications (45). Briefly, MCMV latent mothers were provided *ad libitum* access to water or water containing a combination of four antibiotics ampicillin, neomycin, metronidazole, and vancomycin for 5 days in 1 week. The high-dose antibiotics-treatment group was given 1 mg/mL of ampicillin, 1 mg/mL of neomycin, 1 mg/mL of metronidazole, and 0.5 mg/mL of vancomycin, and the low-dose antibiotics-treatment group was given 0.1 mg/mL of ampicillin, 0.1 mg/mL of neomycin, 0.1 mg/mL of metronidazole, and 0.05 mg/mL of vancomycin. The administration of antibiotics was started 1 week ago of the mating.

### Counting of microorganisms in the cecum

Aerobic and anaerobic bacteria were counted by 10-fold dilution methods. Trypto-soy agar (Eiken Chemical, Tochigi, Japan) was used for aerobic microbes, and GAM agar (Nissui, Tokyo, Japan) was used for anaerobic microbes, respectively. The aerobic cultures were incubated for 24 h at 37℃. The anaerobic microbes were cultured using the AnaeroPack-Anaero system (Mitsubishi Gas Chemical, Tokyo, Japan), and the cultures were incubated for 48 h at 37℃.

### Microbial population analysis

DNA was isolated from the cecum 5 days after antibiotics treatment. The pooled samples were used for the microbial population analysis, as a custom order by Hokkaido System Science Co. (Sapporo, Japan). In brief, a portion of the 16S rRNA gene was PCR amplified and sequenced to characterize bacterial and archaeal community composition. The QIIME pipeline was used for data analysis. Bacterial 16S rRNA sequences were clustered into OTUs at the 97% similarity level.

### CMV reactivation assays

The establishment of reactivation was verified by the detection of MCMV IE1 mRNA in the salivary gland, mammary tissues, and colon. As previously reported (14, 46, 47), total RNA was extracted from 25 mg of a sample using the RNeasy Mini Kit (Qiagen Co., Tokyo, Japan), according to the manufacturer’s instructions. In the first step, cDNA corresponding to 10 µg of RNA was synthesized using the QuantiTect Reverse Transcription kit (QIAGEN), and all the procedures were performed according to the manufacturer’s instructions. This material was a template for the second step of RT-qPCR. MCMV IE1 mRNA was quantified by RT-qPCR using hydrolysis probe chemistry. RT-qPCRs of 20 µL were conducted in the LightCycler 480II instrument: 10 µL of 2× LightCycler 480 probes Master (Roche Diagnostics, Mannheim, Germany), 0.5 µM of final concentration for each primer, 0.2 µM of the final concentration of probe, 5 µL template and distilled water were added to reach the final total volume of 20 µL. RT-qPCR was performed in two steps with the following thermal settings: 5 min at 95°C for initial enzyme activation, followed by 45 amplification cycles (each 10 s at 95°C, 30 s at 60°C, and 1 s at 72°C with fluorescence detection). MCMV-IE1-specific primers and hydrolysis probe were generated as follows: forward, 5′-TGTGTGGATACGCTCTCACCTCTAT-3′; reverse, 5′-GTTACACCAAGCCTTTCCTGGAT-3′; hydrolysis probe, 5'-[FAM] TTCATCTGCTGCCATACTGCCAGCTG [TAMURA]-3'.

pIE III, which contains the full length of the MCMV IE region, was used to generate the standard curve for the quantification of the target gene. Under the conditions described above, the target gene was detected with a linear dynamic range from 10^0^ to 10^6^ copies of MCMV in a sample tube. All test samples, standards, and negative controls were quantified in duplicate.

### Detection of infectious virus

Reactivated pups and uninfected pups were bred with MCMV-reactivation and uninfected mother. After 14 days, reactivated pups and uninfected pups were sacrificed for collection of the salivary gland and colon. The establishment of an infectious virus was verified by the detection of MCMV IE1 mRNA in the salivary gland and colon. In other experiments, an investigation of CMV infectivity titer was performed using median TCID50. A 10% (wt/vol) organ homogenate was prepared in Dulbecco’s modified Eagle’s medium (DMEM)—10% fetal calf serum (FCS)—10% dimethyl sulfoxide, sonicated for 30 s, and then centrifuged to remove cellular debris. The resulting organ homogenates were diluted 1:5 in DMEM—10% FCS and used to infect monolayers of 3T3 cells in a 96-well plate. Monolayers were monitored daily for 3 weeks for signs of CPE, with the medium being changed every 5 days. To confirm that the homogenized organ had no inhibitory effect on viral CPE, 10 PFU of MCMV Smith was added to 50 mL of each sample before incubation on 3T3 cell, and results were compared to those obtained with 3T3 cells infected with 10 PFU of MCMV in medium alone.

### TNF-α mRNA detection assay

TNF-α mRNA detection assay was performed as previously reported with minor modification (47). RNA from the blood, salivary gland, mammary tissues, and colon was extracted using RNeasy Mini Kit (QIAGEN). The procedures of RT-qPCR were performed according to the manufacturer’s instructions. The primers and probes for mouse TNF-α, and GAPDH in RT-qPCR were purchased from Applied Biosystems (TaqMan Gene Expression Assays). RT-qPCR was performed with the LightCycler 480II instrument-like qPCR. The expression level of GAPDH mRNA was used as a reference gene.

### Statistics

The data were expressed as the mean ± SD. The statistical analysis was performed using Student’s *t*-test. *P* value <0.05 was considered to indicate a statistically significant difference.
